# Degree of polymerization and spatial distributions of acyclic and cyclic oligohexoses in soybean root nodules uncovered by MALDI and nanophotonic laser desorption ionization mass spectrometry

**DOI:** 10.1016/j.mtbio.2025.101776

**Published:** 2025-04-18

**Authors:** Chloe Corning, Marjan Dolatmoradi, Tina H. Tran, Gary Stacey, Lajos Szente, Laith Z. Samarah, Akos Vertes

**Affiliations:** a Department of Chemistry, The George Washington University, Washington, DC, 20052, USA; b Divisions of Plant Sciences and Technology, C. S. Bond Life Sciences Center, University of Missouri, Columbia, MO, 65211, USA; c CycloLab Cyclodextrin Research & Development Laboratory, Ltd., H-1097, Budapest, Hungary

**Keywords:** Acyclic oligohexoses, Cyclic oligohexoses, Degree of polymerization, Spatial distribution, Mass spectrometry imaging, Silicon nanopost array (NAPA), Nanophotonic ionization, MALDI, Root nodule, *Bradyrhizobium japonicum*, Soybean, *Glycine max*

## Abstract

In the symbiotic relationship of legumes and rhizobia, disaccharides, mostly sucrose, are produced by the plant and provided as energy and carbon sources for the bacteria. The microbes, in turn, store these carbohydrates as acyclic oligohexoses to buffer fluctuations in supply. Simultaneously, cyclic oligohexoses (β-glucans) of varying sizes and structures are synthesized by nitrogen-fixing soil bacteria both in free living form and in legume root nodules. In the bacteroids, transformed from *Bradyrhizobium japonicum* strain USDA110 in soybean (*Glycine max*) root nodules, glucose units are attached by glycosidic bonds and are known to contain degrees of polymerization with 10 ≤ *n* ≤ 13 repeat units in branched cyclic structures. Whereas cyclic β-glucans (CβGs) are thought to facilitate bacterial adaptation and legume-rhizobia symbiosis, information on their ring sizes, branching from the ring structures, and their spatial distributions within the nodules is scarce. Here we demonstrate that using mass spectrometry (MS), based on matrix-assisted laser desorption ionization (MALDI) and laser desorption ionization (LDI) from emerging silicon nanopost array (NAPA) nanophotonic platforms, the presence of a wider array of potentially cyclic oligohexoses can be discovered with degrees of polymerization in the 2 ≤ *n* ≤ 14 residue range. On the low end of the oligomer size distribution, the cyclic nature of CY_n_ with *n* < 10 can be increasingly questioned based on the large strain such macrocycles would exhibit and the DP control during the CβG synthesis by the glucan phosphorylase involved in their synthesis. At the same time, acyclic oligohexoses with a degree of polymerization of 2 ≤ *n* ≤ 13 were also detected. Tandem MS with collision induced dissociation (CID) indicated that the cyclic structure with *n* = 12 contained a branching residue. It detached from the macrocycle at lower collision energies (70 instrument units), whereas the rings themselves fragmented at higher energies (90 instrument units). We also prove that the spatial distributions of acyclic and cyclic oligohexoses in the *G.max* nodules can be captured by MS imaging (MSI) based on MALDI and NAPA-LDI. The acyclic species were more abundant in the infection zone, whereas the cyclic oligohexoses appeared more concentrated in the inner cortex and in the root vasculature. At some locations, possibly in the vascular bundles surrounding the nodule and traversing the root, the cyclic oligohexoses were especially abundant. The distributions of acyclic oligohexoses were also mapped in the nodule sections. These linear or branching molecules were abundant in the infection zone, where the cyclic oligohexoses were less concentrated or absent.

## Graphical abstract

**Figure:**
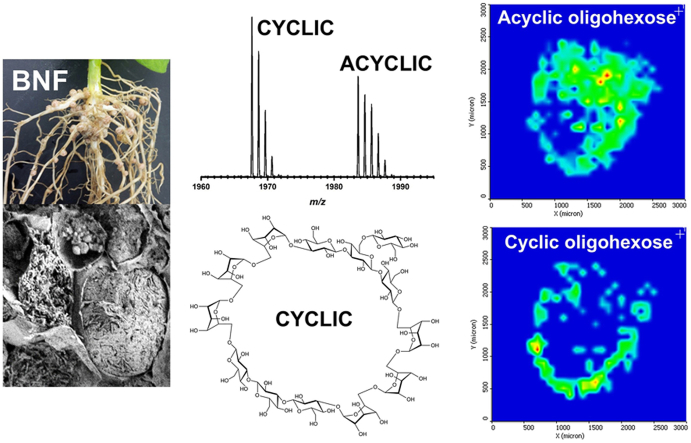

## 1 Introduction

Structurally diverse carbohydrates in symbiotic plant-microbe systems performing biological nitrogen fixation play a multifaceted role. In return for soluble nitrogen containing compounds produced by the bacteria, the host plants provide carbohydrates, e.g., sucrose, as energy and carbon source for the bacteroids functioning in the root nodules [1]. This indispensable resource is stored locally in the form of bio-oligomers, including oligohexoses [2]. More specialized carbohydrates in symbiotic microorganisms play an important role in both bacterial defense and the infection of the host plant [3]. Gram-negative bacteria produce diverse oligosaccharides that serve essential functions within the bacterial cells and in their interactions with the environment [4]. Although the full array of the roles cyclic β-glucans (CβGs) play in the plant cell is unclear, they seem to facilitate mutualistic interactions. Early on, osmoprotection of the bacterial cell in environments with diverse osmolarities was associated with CβGs [5]. As CβGs form inclusion complexes with hydrophobic molecules, e.g., flavonoids, a possible role of CβGs is to shuttle signaling plant metabolites through cell membranes and into aqueous intracellular compartments [6,7]. Another proposed function of CβGs in symbiosis is the suppression of the host plant defense system in response to the bacterial infection [[8], [9], [10]].

In the *Bradyrhizobium* genus, the monomers in CβGs are attached by β-(1,6) and β-(1,3) glycosidic bonds with the degree of polymerization (DP) exhibiting 10 ≤ *n* ≤ 13, where *n* is the number of repeat units [[11], [12], [13]]. Glycosyltransferases present in the membrane of *Bradyrhizobium japonicum* were found to facilitate the biosynthesis of these CβGs [14]. Whereas the enzyme responsible for the synthesis of CβGs with β-(1,2) bonds in other bacteria (e.g., *Agrobacterium tumefaciens*), cyclic glucan synthase (Cgs), has recently been thoroughly characterized [7,15], the corresponding enzyme producing β-(1,6) and β-(1,3) linkages in *B. japonicum* is not fully described.

The CβGs are abundant components of bacterial cells. Their concentrations are estimated to be ∼15 mM in the periplasmic compartment of gram-negative species, including those in the *Rhizobiaceae* family [6,16]. The reported concentrations of CβGs translate into five to twenty percent of the dry weight of bacterial cells [6]. Osmotic adaptation of *B. japonicum* is also linked to the synthesis of β-(1,6), β-(1,3) CβGs [17]. It was found that concentrations of cyclic β-(1,6), β-(1,3) glucans stayed similar between the free-living *B. japonicum* bacteria and the bacteroids performing biological nitrogen fixation in the root nodules of *Glycine max* [6,12,18].

Early structural characterization of CβGs relied on various forms of chromatography (gel filtration, gas chromatography (GC), and HPLC) [5,16,19,20], NMR, [11,18,21], and mass spectrometry (MS) with fast atom bombardment (FAB), [9,11]. The presence of cyclic oligohexose structures in algal species was indicated by matrix-assisted laser desorption ionization (MALDI) ionization MS [22]. Glycosidic linkage analysis was typically performed by multiple chemical transformations (permethylation, acidic hydrolysis, reduction, and acetylation) followed by GC-MS [11,19,23,24]. More recently, advanced MS methods have been used to facilitate the structure determination of CβGs. Electrospray ionization (ESI) tandem MS with collision-induced dissociation (CID) was implemented to distinguish cyclic and acyclic oligosaccharides [25,26]. The DPs for CβGs can be rapidly determined by MALDI-MS analysis of purified bacterial extracts [27,28].

For metabolite analysis and tissue imaging by MS, MALDI is one of the mainstream ion sources [29,30]. A sophisticated three-dimensional mass spectrometry imaging (MSI) based on MALDI and Fourier transform ion cyclotron resonance (FTICR) MS gave further insight into the distribution of numerous metabolites, including carbohydrates and flavonoids in soybean root nodules [31]. More recently, a novel ionization method, laser ablation electrospray ionization (LAESI) was combined with a 21 T FTICR to capture isotopic fine structures for unidentified metabolites in the nodules and discover their elemental formulas [32,33]. Inorganic nanoparticles are gaining importance for biomedical analysis by LDI-MS [34]. In addition, implementing a novel matrix-free nanophotonic ionization method, laser desorption ionization (LDI) from silicon nanopost array (NAPA) platforms, for MSI resulted in complementary molecular coverage and reduced spectral interferences [35]. In direct comparisons for the detection and MSI of metabolites, NAPA provided higher coverage in the low mass region, e.g., for amino acids, whereas MALDI was more efficient in detecting higher mass metabolites, e.g., nucleotides [36,37]. The nanophotonic technique was also used to determine the distributions of bio-oligomers in soybean root nodules. Complementary images of acyclic oligohexoses by NAPA-LDI-MSI and MALDI-MSI revealed a DP exhibiting 2 ≤ *n* ≤ 12 and polydispersity variations within the nodule [2].

Despite accumulating evidence about the structure and function of cyclic oligohexoses, little is known about their spatial distributions and DP variations within root nodules. In this work, we demonstrate that using MALDI-MSI and the emerging nanophotonic NAPA-LDI-MSI method enable the in-depth analysis and imaging of both acyclic and cyclic oligohexoses in soybean root nodules. Our initial findings reveal new evidence on the presence and spatial distributions of a wide array of acyclic and cyclic oligohexoses and their DP in the symbiotic organ responsible for biological nitrogen fixation.

## 2 Materials and methods

### 2.1 Materials

Solvent for the MALDI matrix was prepared with HPLC grade methanol (Prod. No. A452-4, Fisher Scientific) and water (Cat. No. W5-4, Fisher Scientific). High purity 2,5-dihydroxybenzoic acid (DHB), used as a matrix for MALDI-MS (>99.0 % purity, Prod. No. 85707), and 2-propanol (Prod. No. I9516) used for cleaning the oscillating capillary nebulizer were purchased from Millipore Sigma. To prevent the infection of the bacterial cultures, tetracycline (Prod. No. 87128, Millipore Sigma) and spectinomycin hydrochloride (Prod. No. PHR1426, Millipore Sigma) antibiotics were added to the medium.

### 2.2 Culturing *B. japonicum* and producing the *G. max* root nodules

As previously described, wild-type *B*. *japonicum* USDA110 strain was cultured in HM medium for 3 to 4 days in a shaking incubator at 30 °C and 180 rpm, until an optical density of 0.8 at 600 nm corresponding to 10^8^ cells/mL was reached [33]. To protect the culture from infection by competing microorganisms, the medium contained tetracycline and spectinomycin at 25 mg/L and 100 mg/L concentrations, respectively. Pellets of the rhizobia were formed by centrifugation (3000 rpm, 10 min), washed by sterile water, and vortexed to form a suspension for inoculation.

Sterilization, potting and inoculation of the *G*. max (Williams 82) seeds were described in a previous publication [32]. Briefly, sterilized 1:3 perlite:vermiculite potting mix and water were combined until a gel-like texture was reached. Soybean seeds without discoloration, holes, or tears were selected, sterilized using 20 % (v/v) bleach, and washed with water. The seeds were planted in pots containing the potting mix, and 500 μL bacterial suspension was added to each seed. The tray containing the pots was filled halfway with B&D medium used for growing legumes [38]. After developing for 21 days in a growth chamber (Model E36HO, Percival Scientific) with a 16 h/8 h light/dark cycle at 30 °C, the root nodules were harvested, snap-frozen in liquid nitrogen, and stored in a −80 °C freezer.

### 2.3 Mass spectrometry and molecular imaging

As described in previous publications, in order to prepare the soybean nodules for MSI, 10 μm-thick sections were cut and mounted on a microscope slide for MALDI or on a NAPA imaging chip by a cryomicrotome (CM1800, Leica Microsystems Inc., Nussloch, Germany), followed by 30-min drying in a desiccator [36]. For MALDI, a home-built oscillating capillary nebulizer was used for the application DHB matrix to the nodule section with a 10-s on and 30-s off cycle until the sample was evenly coated. To achieve uniform coating, the oscillating capillary was positioned ∼23 cm above the tissue surface and spraying was driven by nitrogen at a pressure of 276 kPa [36]. After the matrix application, the sample was placed back into the desiccator for another 30 min. A thorough description of the preparation of root nodule sections for NAPA-LDI-MSI is given in Ref. [2].

For MSI, a MALDI-LTQ-Orbitrap XL mass spectrometer (Thermo Scientific, San Jose, CA, USA) was used with a nitrogen laser for LDI. The MS resolution was set to 30,000 at *m/z* 400 (FWHM). In the MALDI imaging experiments, 10 laser shots with ∼7 μJ energy/pulse were focused to 80 μm × 100 μm spots for each pixel. This corresponded to ∼90 mJ/cm^2^ fluence. For NAPA, 10 shots were fired per pixel and the fluence was ∼120 mJ/cm^2^. Positive ion mode mass spectra were recorded pixel by pixel with a raster step size of 100 μm, and false color images were constructed by mapping the intensities for the peaks of interest to the pixel locations on the sample surface using the ImageQuest software (ImageQuest Version 1.0.1, Thermo Scientific, San Jose, CA, USA) [2,35,36].

To enhance molecular coverage, LDI from silicon NAPA was also performed [37]. The fabrication of the nanophotonic NAPA platform was described in detail elsewhere [39]. Briefly, patterns needed to produce the NAPA were created by deep UV projection lithography (DUV-PL) on the surface of oxidized low resistivity silicon wafers. This resulted in a SiO_2_ hard mask that marked the position of the posts. To develop the nanoposts, deep reactive ion etching (DRIE) was implemented with an etching gas mixture of C_4_F_8_, SF_6_, and Ar. Earlier optimization of the NAPA geometry indicated a maximum ion yield for structures with 337 nm periodicity, and 150 nm and 1100 nm post diameter and height, respectively [40,41].

For structural characterization of the cyclic and acyclic oligosaccharides, tandem MS with collision-induced dissociation (CID) was performed at different normalized collision energies [25]. Ultra-high purity He was used as the collision gas at a pressure of 40 ± 10 psi. An isolation window of *m/z* ±1.0, activation q of 0.250, and activation time of 30.000 ms were used for tandem MS.

Recently molecular imaging of bio-oligomers (polyhydroxybutyric acid (PHB), polyglutamic acid (PGA), and polysaccharide oligomers) by MALDI MSI and NAPA MSI have been reported in plant tissues [2]. In this study, for MS and MSI data acquisition, the same protocol was followed, except for the mass spectral acquisition range that was set to 200 ≤ *m/z* ≤ 2500 to capture the peaks corresponding to some of the larger oligohexoses. The automatic gain control (AGC) was turned off for all the experiments and the injection time (IT) was 50 ms. The collected mass spectra were processed using the Xcalibur software (Version 3.0.63; Thermo Fisher Scientific Inc., Bremen, Germany). To find potential matches for the detected metabolites and their pathways, the BioCyc (https://biocyc.org/) and MetaCyc (https://metacyc.org/) databases were searched.

### 2.4 Optical and scanning electron microscopy imaging

**Fig. 1:**
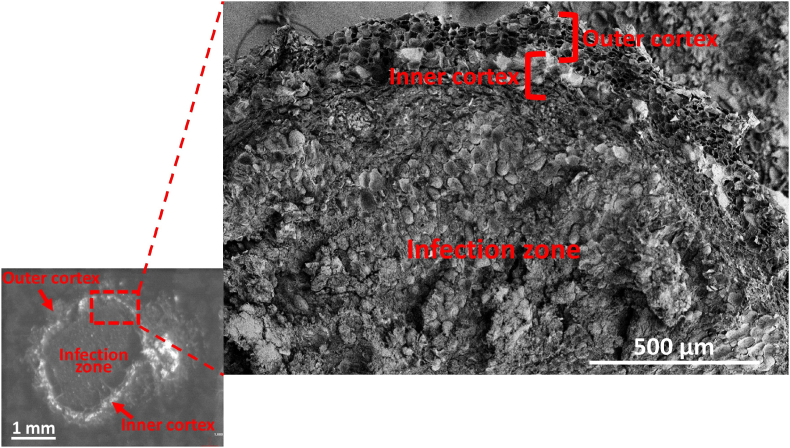
Optical (left) and scanning electron microscope (right) images of a soybean root nodule section. The outer and inner cortex, and the infection zone are marked in both images.

The optical images of the root nodule sections were acquired in the MALDI-LTQ-Orbitrap XL mass spectrometer through its built-in imaging system (see left panel in Fig. 1). This enabled the co-registration of the optical images with the ion distribution maps constructed from the peak intensities in the mass spectra.

To obtain finer details of the nodule structure, i.e., the outer cortex, inner cortex, and the infection zone with cellular resolution, scanning electron microscopy (SEM) (FEI Teneo LV SEM, Thermo Fisher Scientific, Hillsboro, OR, USA) was used. Preparation of the root nodule sections for SEM was described earlier in detail [33]. The walls of the hollowed cells in the inner and outer cortex, and the infected and uninfected cells in the infection zone are clearly discernable in the SEM image (see right panel in Fig. 1).

## 3 Results and discussion

### 3.1 Simultaneous detection of acyclic and cyclic oligohexoses in root nodules

In an earlier study, we detected an array of oligohexoses with 2 ≤ *n* ≤ 12 and imaged their spatial distributions in soybean root nodules by LDI from a NAPA platform [2]. The oligomers were identified based on a *Δm/z* = 162.053 periodicity in the spectra, corresponding to a C_6_H_10_O_5_ repeat unit, stemming from hexose condensation. Imaging by MALDI-MSI with DHB matrix, deposited on the tissue section by sublimation, was unsuccessful [2].

**Fig. 2:**
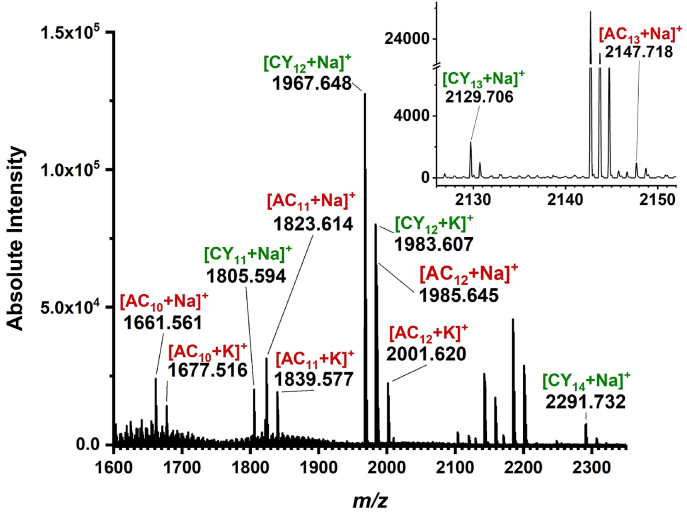
Positive ion MALDI mass spectrum from root nodule section showing *m/z* values for sodiated and potassiated acyclic (AC_n_, where 10 ≤ *n* ≤ 13) and cyclic (CY_n_, where 11 ≤ *n* ≤ 14) oligohexoses with repeat units of C_6_H_10_O_5_. Accurate masses (see Table 1) allowed the distinction between acyclic and cyclic oligohexoses with identical repeat units.

In order to capture a wider range of oligomers and simultaneously detect acyclic and cyclic oligohexoses, MALDI with a different matrix deposition method was implemented. This time the DHB matrix for MALDI was deposited onto the tissue section by an oscillating capillary nebulizer. Positive ion MALDI mass spectra from root nodule sections were acquired from 10 laser pulses of 7 μJ energy each. They showed *m/z* values for sodiated and potassiated acyclic (AC_n_ where 10 ≤ *n* ≤ 13) and cyclic (CY_n_ where 11 ≤ *n* ≤ 14) oligohexoses with repeat units of C_6_H_10_O_5_ (see Fig. 2). Their accurate masses (see Table 1) allowed the distinction between acyclic and cyclic species with identical repeat units. Some doublets, observed in Fig. 2, originate from the simultaneous presence of sodiated and potassiated forms of the same oligohexose species. Similar Na^+^/K^+^ doublets are observed for several ions in this spectrum. To our knowledge, this is the first time cyclic oligohexoses have been detected directly in root nodules by local MS analysis.

**Table 1:** (Top table) Calculated and measured *m/z* values for sodiated **cyclic** oligohexoses determined by MALDI-MS and NAPA-LDI-MS show complementary coverage. (Bottom table) Calculated and measured *m/z* values for sodiated **acyclic** oligohexoses determined by MALDI-MS and NAPA-LDI-MS show complementary coverage.

***n*****(cyclic)**	**Formula**	***m/z***_**calc.**_**[M+Na]**^**+**^	***m/z***_**MALDI**_	***m/z***_**NAPA**_
2	C_12_H_20_O_10_	347.095		**347.096**
3	C_18_H_30_O_15_	509.148		**509.151**
4	C_24_H_40_O_20_	671.201		**671.205**
5	C_30_H_50_O_25_	833.254		**833.262**
6	C_36_H_60_O_30_	995.307		**995.307**
7	C_42_H_70_O_35_	1157.360		**1157.359**
8	C_48_H_80_O_40_	1319.412		**1319.430**
9	C_54_H_90_O_45_	1481.465		**1481.466**
10	C_60_H_100_O_50_	1643.518		**1643.521**
11	C_66_H_110_O_55_	1805.571	**1805.594**	**1805.573**
12	C_72_H_120_O_60_	1967.624	**1967.648**	**1967.626**
13	C_78_H_130_O_65_	2129.676	**2129.706**	
14	C_84_H_140_O_70_	2291.729	**2291.732**	

				
***n* (acyclic)**	**Formula**	***m/z***_**calc.**_**[M+Na]**^**+**^	***m/z***_**MALDI**_	***m/z***_**NAPA**_

2	C_12_H_22_O_11_	365.106		**365.108**
3	C_18_H_32_O_16_	527.159		**527.161**
4	C_24_H_42_O_21_	689.212		**689.214**
5	C_30_H_52_O_26_	851.264		**851.271**
6	C_36_H_62_O_31_	1013.318		**1013.321**
7	C_42_H_72_O_36_	1175.371		**1175.374**
8	C_48_H_82_O_41_	1337.423		**1337.431**
9	C_54_H_92_O_46_	1499.476		**1499.489**
10	C_60_H_102_O_51_	1661.529	**1661.561**	
11	C_66_H_112_O_56_	1823.582	**1823.614**	
12	C_72_H_122_O_61_	1985.635	**1985.645**	
13	C_78_H_132_O_66_	2147.687	**2147.718**	
14	C_84_H_142_O_71_	2309.740		

Significantly, this capability enables the exploration of spatial distributions of these species within the root nodule. Although the nature of the glycosidic bond is not revealed by the MALDI-MS data, one can hypothesize that at least some of the cyclic species are CβGs. The relatively high ∼15 mM concentration of some CβG species in the periplasmic compartment of gram-negative bacteria makes their presence in the spectra likely. Despite the partial success of detecting both acyclic and cyclic oligohexoses by MALDI-MS using the oscillating capillary matrix deposition method, the detection of species with lower number of repeat units, *n* < 10, was not achieved.

**Fig. 3:**
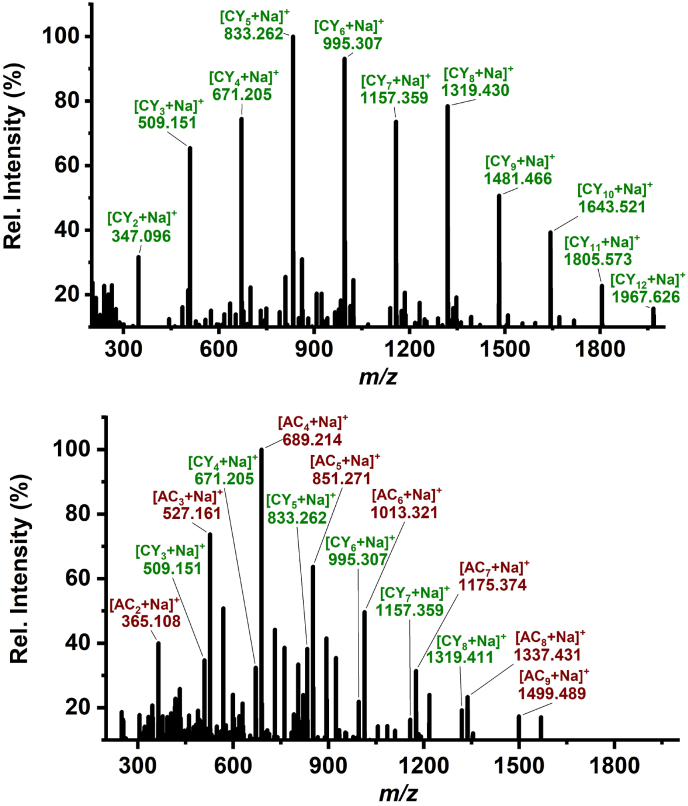
Positive ion NAPA-LDI mass spectra showing (top panel) cyclic oligohexoses, and (bottom panel) both cyclic and acyclic oligohexoses in different locations within a root nodule section. (Data for top panel is from Ref. [2] © 2021 Wiley-VCH GmbH.).

Based on earlier work that showed the detection of oligohexoses in the root nodules by NAPA-LDI-MS [2], this matrix-free method was implemented. A 10 μm-thick section of the soybean root nodule was mounted on a silicon NAPA platform and positive ion mass spectra were taken in the 200 ≤ *m/z* ≤ 2000 range by LDI with ∼110 mJ/cm^2^. Depending on the location in the nodule where the spectra are collected, one can observe either a single set of oligohexoses, e.g., only the cyclic oligohexoses seen in the top panel of Fig. 3, or two interleaved series of peaks representing both the cyclic and acyclic species (see the bottom panel in Fig. 3). Both series exhibit a *Δm/z* = 162.05 periodicity indicating a C_6_H_10_O_5_ repeat unit. They are, however, shifted by *Δm/z* = 18.01 with respect to each other, consistent with the loss of H_2_O, from cyclization via a condensation reaction. The sodiated acyclic oligohexoses, marked with red in the spectrum, exhibit a DP with 2 ≤ *n* ≤ 9, whereas the cyclic species in the top panel, marked with green, are present with 2 ≤ *n* ≤ 12 repeat units.

Cyclic structure for the larger oligomers, 4 ≤ *n* is a distinct possibility, however, for *n* < 4, increased strain in the macrocycle makes ring formation less likely. Although small cyclic oligosaccharides have been synthesized in the laboratory [13], e.g., the peracetylated versions of cyclogentiobiose [[42], [43], [44]], cyclogentiotriose [45,46], and cyclogentiotetraose [[42], [43], [44]], and small cyclodextrins as cyclic α-1,4-D-glucopyranoside trimers and tetramers [47], their occurrence in nature has not been reported. The mechanism of CβG production by the Cgs enzyme is envisioned through four steps: initiation by Cgs autoglycosylation, elongation by addition of glucose from UDP-Glc, control of the DP by a glucan phosphorylase in the C-terminal region of Cgs [48], and cyclization [7,15]. Due to the length control step and the increasing strain affecting cyclization, the formation of small CβGs in nature is unlikely. Alternative explanations for the presence of dehydrated ions isomeric with [CY_n_ + Na]^+^ are in-source water loss from the acyclic sodiated oligohexoses without cyclization [AC_n_-H_2_O + Na]^+^, or the presence of acyclic anhydro oligosaccharides, an unlikely option because they are typically formed by pyrolysis [49]. Collision-induced dissociation (CID) of sodiated monosaccharides and quantum chemical calculations indicate that dehydration reactions leading to [AC_1_-H_2_O + Na]^+^ are possible [50,51]. It is unclear if this reaction channel is active under LDI conditions and for larger DP ions.

Both MALDI and NAPA-LDI have limitations for high molecular weight biooligomers. In a previous study we compared the performance of the two techniques for the analysis of synthetic poly-3-hydroxybutyric acid (PHB) standards, with a number-average molar mass of M_n_ = 2000 Da determined by gel permeation chromatography [2]. Both MALDI-MS and NAPA-LDI-MS were able to detect PHB oligomers up to *m/z* ≤ 4000 with M_n_ = 1584 Da and M_n_ = 1200 Da, respectively. Although oligohexoses probably have lower ionization yields than PHBs, the detected oligohexoses were all below *m/z* 2500 with no hint of abundant species in the 2500 ≤ *m/z* ≤ 4000 range. To improve the sensitivity of these techniques for higher *m/z* oligomers, improved sample preparation techniques, different matrixes for MALDI, and optimized nanostructures for NAPA-LDI can be explored.

### 3.2 Tandem MS for structural characterization

**Fig. 4:**
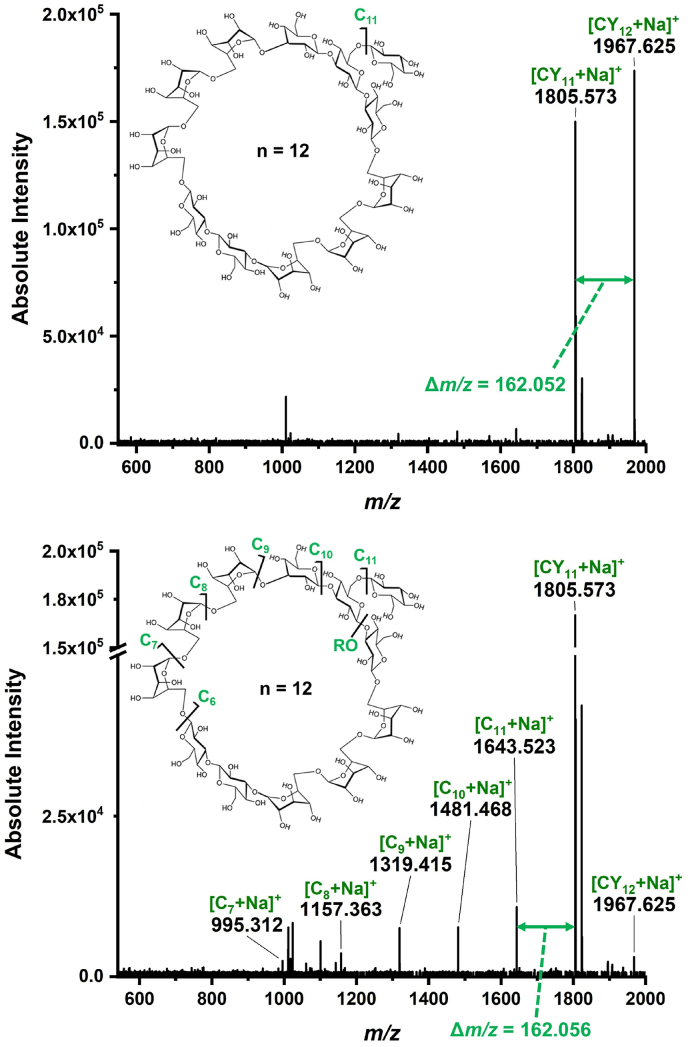
Tandem mass spectra of sodiated *n* = 12 oligohexose with nominal *m/z* 1967.6 precursor from nodule section by CID (top panel) at 70 instrument unit and (bottom panel) at 90 instrument unit collision energies. Fragmentation patterns are consistent with a cyclic structure of 11 hexose units and a branching substituent of a single hexose (i.e., *n* = 11 + 1) shown in the insets. RO indicates a potential ring opening site.

Based on the prevalence of large CβGs in soil bacteria, the presence of macrocycles is a clear possibility. However, the existence and nature of substituents on the ring structure are unclear. To explore the structure of larger oligohexoses, CID-based tandem MS with varying collision energies was performed on the *m/z* 1967.648 ion (*n* = 12 repeat units) produced by MALDI. In Fig. 4, the top and bottom panels show the fragmentation at 70 and 90 collision energies in instrument units, respectively. Although the loss of C_6_H_10_O_5_ repeat unit is obvious in both spectra, the naming of the fragments is not straightforward due to the unresolved debate about the applicability of the conventional oligosaccharide fragmentation nomenclature [52] to macrocyclic structures [26,[53], [54], [55]]. The root of the dilemma is the lack of reducing and non-reducing ends for cyclic structures, central to the original naming convention.

Irrespective of the nomenclature, the fragmentation patterns are consistent with a cyclic structure of 11 hexose units and a substituent of a single hexose shown in the insets. For 90 instrument unit collision energy, the relative positions of the ring opening (RO) site and the substituent are not revealed by the CID data. Indeed, it is also possible that the *m/z* 1967.648 precursor is an in-source fragment of the corresponding acyclic dodeca-hexose sodium adduct. The loss of a single hexose unit at 70 instrument unit collision energy, however, makes the branched ring structure more likely. In *B. japonicum* USDA 110, the presence of a similar highly abundant CβG macrocycle with a ring of 12 glucose residues and a single branching glucose substituent had been demonstrated by end group analysis, HPLC, MS, and NMR studies [6,9,18].

### 3.3 Spatial distributions of cyclic and acyclic oligohexoses

**Fig. 5:**
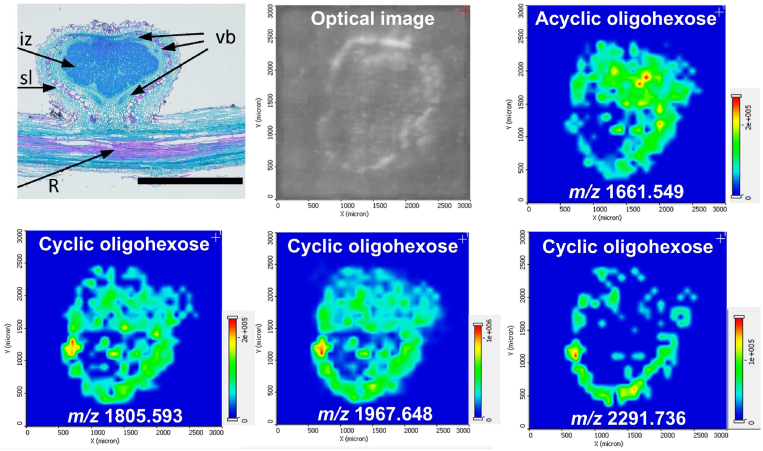
Anatomy of a soybean root nodule section (top left) (scale bar 1 mm) based on a transmission microscope image, reflection optical image of the studied section in mass spectrometer (top middle), corresponding ion images for acyclic (top right) and cyclic (bottom) sodiated oligohexoses captured by MALDI-MSI. The acyclic species is concentrated in the infection zone, whereas the cyclic oligohexoses are more abundant in the inner cortex. High intensity spots within the cortex might be associated with vascular bundles. Arrows in the top left panel point out the root vasculature (R), vascular bundle (vb) within the inner cortex, sclereid layer (sl) in the outer cortex, and the infection zone (iz). (Top left panel is adapted from Ref. [56].).

Fig. 2 showed the presence of a mixture of acyclic and what appears to be cyclic oligohexoses with DPs of 10 **≤** *n* ≤ 13 and 11 **≤** *n* ≤ 14, respectively. Our tandem MS results in Fig. 4 and extensive literature data on CβG in some Gram-negative soil bacteria support the idea that the CY_n_ ions in Fig. 2 indeed originate from macrocycles. Based on the strong signal for both AC_n_ and CY_n_ ions, the distributions of the related molecules can be explored by MALDI-MSI. In Fig. 5, the anatomy of the root nodule is presented alongside the spatial distributions of an acyclic and three cyclic oligohexoses. In the top left panel, the tangential section of the root-nodule interface is depicted [56]. Safranin O is used to stain the lignified cells in the sclereid layer (sl) of the outer cortex, and fast green FCF is applied to stain cell walls throughout the entire section. This reveals the anatomy of the nodule with respect to vasculature and enables the distinction of the root vasculature (R), the vascular bundle (vb) within the inner cortex, and the infection zone (iz). In the top middle panel of Fig. 5, the optical image of the matrix covered nodule section is shown in the mass spectrometer.

The top right panel in Fig. 5 displays the distribution of [AC_10_+Na]^+^ ions originating from the acyclic AC_10_ species in the tissue. The most significant abundances of this molecule, appearing as orange and red hotspots, are localized to the infection zone within the nodule. This is consistent with the role of acyclic oligohexoses as energy and carbon storage molecules.

Imaging the potentially cyclic oligohexose species resulted in significantly different distributions. The three bottom panels, corresponding to the sodiated form of CY_11_ (*m/z* 1805.593), CY_12_ (*m/z* 1967.648), and CY_14_ (*m/z* 2291.736) indicate lower or no abundance for these species in the infection zone, and significant presence in the inner cortex. The highest abundances, appearing as orange and red hotspots, are also found in this layer for all three species. Comparing these images to the top left panel, these maxima can be hypothetically attributed to the vascular bundles encapsulating the infection zone. The fundamentally different patterns of AC_n_ and CY_n_ distributions, i.e., the very low level of colocalization, also make it less likely that for *n* < 10 the ionic CY_n_ species are exclusively the products of in-source fragmentation of AC_n_ ions.

**Fig. 6:**
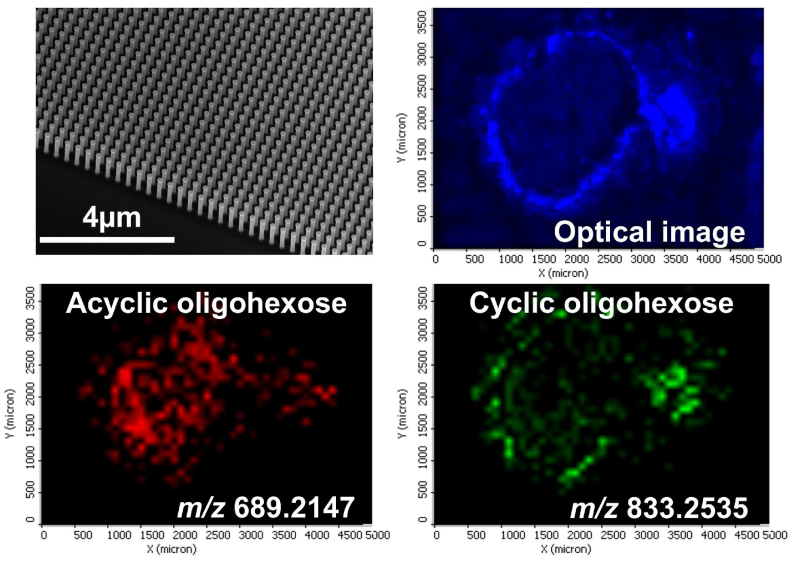
NAPA-LDI-MS imaging of acyclic and cyclic oligohexoses at the root-nodule interface. The top left panel shows the SEM image of the silicon NAPA nanostructure before mounting the tissue section. Optical image of the tissue section in the mass spectrometer shows the blue false color image of the cortex and the root cross section (top right panel). Spatial distributions of acyclic (marked with red false color in bottom left panel) and cyclic oligohexoses (marked with green false color in bottom right panel) in the transverse section of the soybean root-nodule interface achieved by NAPA-LDI-MSI in positive ion mode. The acyclic oligohexose is mostly found in the infection zone, whereas the cyclic oligohexose resides in the inner cortex and the root. High abundance areas for the cyclic molecules appear to be associated with the vasculature surrounding the nodule and are prevalent in the root. (For interpretation of the references to color in this figure legend, the reader is referred to the Web version of this article.)

To explore the colocalization of smaller AC_n_ and CY_n_ species, their distributions were determined by NAPA-LDI-MSI. In the top left panel of Fig. 6, the SEM image of the silicon NAPA platform is shown. Transverse tissue sections of the root-nodule interface were directly mounted on these structures and transferred into the mass spectrometer for optical and molecular imaging. The reflective optical image of the section is shown in the top right panel highlighting the cortex of the nodule and the cross section of the supporting root. Distributions of the sodiated AC_4_ and CY_5_ acyclic and cyclic oligohexose species are displayed in the lower left and lower right panels of Fig. 6, respectively. Similar to the MALDI images of larger homologues, the acyclic species is more abundant in the infection zone, whereas the cyclic species shows higher levels in the cortex of the nodule and in the adjacent root. Regarding reproducibility, it was reassuring that the acyclic oligohexoses with different degrees of polymerization all showed elevated abundances in the infection zone. Likewise, cyclic oligohexoses with different numbers of repeat units all showed higher abundances in the inner cortex (see, e.g., the *n* = 10 distribution in the top right panel of Fig. 5). This observation for MALDI-MS images of cyclic oligohexose distributions with *n* = 11, 12, and 13 is demonstrated in the three bottom panels of Fig. 5. Acquired by the emerging matrix-free NAPA-LDI-MS method, the images in Fig. 6 show a similar difference in the distributions of acyclic (*n* = 4) and cyclic (*n* = 5) oligohexoses.

The difference in the spatial distributions of the acyclic and cyclic species seems to correlate with the presumed role of these two types of molecules as energy and carbon storage for the acyclic species and signaling between the bacteria and the plant for the cyclic series. In the symbiotic relationship, the plant provides energy and carbon, mostly in the form of sucrose to the bacteroids in the infection zone. There, the surplus can be stored as acyclic oligosaccharides for later use. The cyclic oligohexoses, e.g., CβGs to suppress the host plant defense system, are brought to the nodule by the infecting bacteria and distributed to the plant through the vasculature, including the vascular bundles in the inner cortex and the vasculature in the roots. However, due to DP control during the CβG synthesis by a glucan phosphorylase and the elevated strain in small macrocycles, for *n* < 10 the [CY_n_ + Na]^+^ ions originating from naturally occurring CY_n_ are gradually replaced by [AC_n_-H_2_O + Na]^+^ produced by in-source water loss from acyclic sodiated oligohexoses.

## 4 Conclusions

The occurrence, DP, and spatial distributions of oligohexoses in the root nodules of soybean infected by *B. japonicum* have been studied by MALDI-MSI and NAPA-LDI-MSI. In a departure from earlier findings, wide arrays of both acyclic, AC_n_, and what appear to be cyclic, CY_n_, species have been detected. The acyclic molecules exhibited a DP with 2 ≤ *n* ≤ 13, whereas the cyclic species and their isomers derived by in-source fragmentation, oligomers with 2 ≤ *n* ≤ 14 were found. In previous studies, CβGs with 10 ≤ *n* ≤ 13 DP were identified in *B. japonicum* extracts. Our results for the corresponding bacteroids in soybean root nodules extended this on both the high and especially the low end of the oligomer size distribution. Two ionization techniques, MALDI and NAPA-LDI, indicated the simultaneous presence of the acyclic and cyclic species. Alternatively, the formation of structural isomers of the CY_n_ ions from AC_n_ ions by in-source water loss, [AC_n_-H_2_O + Na]^+^, or the presence of anhydro oligosaccharides in the nodule tissue can also explain the formation of [(C_6_H_10_O_5_)_n_ + Na]^+^ ions. On the low end of the oligomer size distribution, the cyclic nature of CY_n_ with *n* < 10 can be increasingly questioned based on the large strain such macrocycles would exhibit and the DP control during the CβG synthesis by the glucan phosphorylase involved in their synthesis.

Similarly to earlier studies on hepatocyte extracts and human urine, spectral complementarity was found between MALDI-MS and NAPA-LDI-MS results [37]. The oligosaccharides detected by the latter covered the 2 ≤ *n* ≤ 12 DP range with lower molecular weight and diminishing ion intensities for *m/z* > 1800, whereas MALDI produced strong signal for 10 ≤ *n* ≤ 14 oligomers. Although numerous *Proteobacteria* are known to contain high concentrations of cyclic β-1,2-D-glucans composed of 17–25 glucose units [7], in our study involving *B. japonicum* no oligomers were observed with *n* > 14 DPs. It is unclear if larger oligomers are truly absent from the nodule tissues or the sensitivity of MALDI for their direct detection in the tissue declines for those higher *m/z* values. In future studies, MALDI or ESI mass spectra of tissue extracts can be compared to local analysis directly from the tissue to resolve this question.

Our study revealed differences in the localization of acyclic and cyclic oligohexoses within the nodule. Whereas acyclic species were more abundant in the infection zone, the cyclic molecules exhibited higher concentrations in the inner cortex, and potentially in the vasculature. It was hypothesized that the specific distributions were linked to the function of these molecular classes, i.e., energy and carbon storage for the acyclic and signaling and immunomodulation for cyclic oligohexoses. Further work is needed to test the above hypothesis and more precisely verify the localization of these molecules. Such studies can include genetic modification of the enzyme responsible for glucan cyclization in B. japonicum [15]. Current advances in tissue embedded single cell analysis present an additional opportunity to gain deeper insight into their role within the nodule [57].

## CRediT authorship contribution statement

**Chloe Corning:** Writing – original draft, Formal analysis, Data curation. **Marjan Dolatmoradi:** Formal analysis, Data curation. **Tina H. Tran:** Formal analysis, Data curation. **Gary Stacey:** Writing – review & editing, Conceptualization. **Lajos Szente:** Writing – review & editing, Conceptualization. **Laith Z. Samarah:** Writing – review & editing, Formal analysis, Data curation. **Akos Vertes:** Writing – review & editing, Writing – original draft, Supervision, Project administration, Methodology, Investigation, Conceptualization.

## Funding statement

This work was supported, in part, by the 10.13039/100000001U.S. National Science Foundation, Plant Genome Program; 10.13039/100000154Division of Integrative Organismal Systems (Grant Number: IoS-1734145) awarded to G.S.

## Declaration of competing interest

The authors declare that they have no known competing financial interests or personal relationships that could have appeared to influence the work reported in this paper.

## Data Availability

The data supporting the findings of this study are available from the corresponding author upon request.
